# Electronic Documentation Practice and Its Correlated Factors Among Medical-Surgical Nurses in Selected Hospitals in Texas, USA

**DOI:** 10.7759/cureus.100924

**Published:** 2026-01-06

**Authors:** Ma. Theresa M Popatco, Alfredo Z. Feliciano

**Affiliations:** 1 Graduate School, Angeles University Foundation, Angeles, PHL

**Keywords:** correlated factors, electronic nursing documentation, knowledge and attitude, organizational factors, practices

## Abstract

Introduction: Nursing documentation provides evidence of the care delivered by clearly accounting for what occurred and when it occurred. Despite the overall importance of electronic documentation, available evidence shows significant limitations in good practice.

Objective: This study aimed to describe the electronic documentation practice and its correlated factors among medical and surgical ward nurses in four selected hospitals in Texas, USA.

Methods: The descriptive correlational study involved 379 medical-surgical nurses recruited through proportionate non-probability sampling from four hospitals in Texas, USA, with the intent of examining how socio-demographic, knowledge, attitude, and organizational factors influence electronic documentation practices. Data collected through an online questionnaire were analyzed using descriptive statistics, Spearman rho correlation, and linear regression to determine relationships and factors associated with documentation practices.

Results and discussion: Most respondents were female (244, 64.4%), married (186, 49.1%), held a bachelor’s degree (289, 76.3%), and were employed in general hospitals (254, 69%) and medical wards (290, 76.5%) for an average of 9.77 years (SD±8.51). All nurses exhibited good knowledge in areas of communication, legal protection, and patient safety, and the majority (369, 97.4%) demonstrated a favorable attitude toward the legal and clinical significance of electronic documentation. Documentation practices were strong in recording assessments, interventions, and medication administration, while lower mean scores were noted in documenting nursing diagnoses, patient education, and immediate post-care entries. Institutional supports included in-service training, operational standards, available documentation materials, and supervisory follow-up. However, more than 80% (318) reported fatigue and exhaustion, and fewer than 20% (64) indicated a lack of time and motivation for electronic documentation. Documentation practices were positively associated with educational attainment, knowledge, attitude, and organizational support (p < .01), while gender, age, income, hospital type, and years of experience showed no statistically significant correlation. Divorced nurses had poorer documentation scores compared to other marital groups.

Conclusion and recommendations: High knowledge and favorable attitude, when supported by strong organizational systems, contribute to good documentation practices among nurses. The study recommends strengthening in-service training, addressing work-related fatigue, enhancing supervisory support and monitoring, and promoting timely documentation.

## Introduction

The nursing process is directed by the implementation of planned interventions delivered to patients under the direction of a qualified nurse [1], clearly accounting for what occurred and when it occurred, making it an important aspect of professional practice [2]. It becomes the standard way of keeping ongoing patient care information [3]. Without a complete recording, there is no evidence that care is provided to the patient [4].

Nursing documentation is the primary source of clinical information to satisfy safe, legal, and clinical standards in patient care [5]. Accurate and complete documentation is essential for ensuring safe, high-quality patient care and protects the health care professionals by providing a clear record of intervention [6]. The quality of care provided to the patients is dependent on the accurate, timely, and effective communication of information with patients and colleagues. It determines the standard and quality of care rendered to the patients [2]. Standard of care is established when the information is communicated across providers, thereby supporting safe and evidence-based nursing practice [7]. Nursing documentation can be classified as electronic and traditional [8]. Paper-based documentation is replaced by electronic documentation due to technological influence in improving the efficiency of recording delivered nursing interventions. 

With the gradual move from paper-based to electronic nursing documentation, challenges can potentially affect accurate practices of documentation to indicate patient safety, quality care, and protected legal implications [9]. The demands of accurate documentation span nurses from all hospital departments. High-quality medical and surgical documentation among nurses is required as they are involved with the care of transitioned patients from intensive care units. Amidst increasing cases of surgical site infections, accurate documentation becomes a professional responsibility as it plays a vital role in the delivery of effective, safe, evidence-based health care and in quality improvement [10].

Similarly, comprehensive nursing care documentation becomes a valuable source of data for health research, evidence, and rationale for funding and resource allocation [4]. In this way, the quality of documentation will benefit the patient, healthcare professionals, and the institution in various ways, inclusive of effective clinical communication, maintenance with accreditation standards, legal and legislative protection, and opportunities for research and quality activities [7].

However, the World Health Organization reported that poor communication between healthcare professionals has become a significant factor for medical errors [1]. Despite the overall importance of nursing documentation, available evidence shows significant limitations in proper practice [7]. When a patient files a complaint for care that was received or not, nursing records become legal proof of care delivery [6]. Therefore, clinical documentation is a part of the healthcare professional's obligation [3].

Despite the evident importance of nursing documentation, time spent on documentation can become substantial. While it varies internationally, nurses spend about 17%, 25%, and 26- 41% of their time in Great Britain, USA, and Canada, respectively [11]. With a heavy clinical workload, erroneous entries may result.

Knowledge and attitude towards documentation among nurses predict the accuracy of clinical recording of provided care. Factors contributing to poor documentation practice of nurses range from inadequacy of knowledge regarding its importance, patient load, lack of training, and support from nursing management, and lack of professional standards and structures for documentation [1,5]. Other factors such as age, educational status, and availability of documenting software/sheets are also seen to relate to nursing documentation practice [6].

Additionally, the quality of documentation is the primary tool to continually measure performance outcomes against predetermined standards of nurses and the organization [12]. Poor documentation is a reflection of deficiencies in care delivery, which can adversely affect patient outcomes, professional accountability, and organizational workflow [5,7]. Moreover, undesirable results emerge, such as longer hospital stays, poor communication between health team members, increased medical risks, impeded clinical decisions, and poor patient care from poor documentation practices [4].

Although several studies have been conducted to determine documentation practices among nurses, issues concerning poor documentation remain relatively high. Additionally, medical-surgical nurses’ practices of documentation are limited in the literature. The time spent by medical-surgical nurses was focused on documentation for each shift [13]. Most studies focused on nurses in general but had not concentrated on medical-surgical nurses. Considerably, electronic documentation practices become the study’s focus because technology moves the practice of documentation among nurses into a more efficient yet challenging one, including assessment and implementation [14,15]. Understanding the selected factors of this problem provides a better understanding and direction for improving nursing documentation as an overall process and task. 

The study aimed to determine the electronic documentation practice and its correlated factors among medical-surgical nurses in selected hospitals in Texas, USA. Specifically, the study aimed to describe the respondents’ socio-demographic and work characteristics, describe their knowledge, attitude, and practices on electronic nursing documentation, assess the organizational factors at work on electronic nursing documentation, and examine how these factors relate to documentation practices.

## Materials and methods

Study design and locale

The study was descriptive correlational in design because it aimed to describe the electronic documentation practice of medical surgical nurses as influenced by identified factors (socio-demographic and work profile, knowledge, attitude, and organizational factors at work on electronic nursing documentation). With the available data on the nurses’ socio-demographic, work, and organizational characteristics, the correlation of these factors with nurses’ electronic documentation practice was determined.

This study was conducted among nurses of the selected hospitals in Texas, USA. This region has a large and diverse healthcare workforce, allowing for a potentially more generalizable sample. The four selected hospitals can potentially represent better generalizability of future findings because of an adequate sample size from a densely populated healthcare workforce in the region. It included four hospitals in Texas with nurse participation as follows-Hospital A (131), Hospital B (90), Hospital C (52), and Hospital D (106), providing a total of 379 respondents and improving transparency regarding site contributions and sample distribution.

Study respondents

Determined using Raosoft sample size computation, a total of 379 respondents was the minimum number required (5% margin of error, confidence level of 95%). After setting the required inclusion criteria, the recruitment of required samples was carried out using a non-probability proportionate sampling to complete the required minimum sample for the study. Based on the available figures (estimated number of hospital nurses), proportionate sampling requires 185 nurses in Hospital A (49%), 75 nurses in Hospital B (20%), 82 nurses (22%), and 37 nurses in Hospital D (10%). Unfortunately, the researcher was not given the specific number of medical surgical nurses present in each hospital and used proportionate sampling according to the population of nurses. 

To be included in the study, the following eligibility characteristics must be present: (a) currently works in the identified hospital for at least three months, (b) registered nurse practicing in the medical or surgical ward in direct patient care, and (c) expresses consent to participate in the study. However, the study excluded those who were on leave or absent at the time of data collection, and those who did not express consent to participate in the study.

Research instruments 

The research instrument was divided into five parts that detail the respondents’ socio-demographic and work characteristics, nursing documentation practice, knowledge and attitude towards nursing documentation, and organizational factors at work. The questionnaire was prepared in electronic form through Google Form, where the link was sent via available modes of communication (e.g., email messaging). The standardized instruments were adopted directly from previously published studies without formal cultural adaptation procedures, and this is now noted as a limitation. Only pilot testing for usability within the electronic survey platform was conducted, and reliability coefficients from the current sample were recalculated to support their use in this context.

Sociodemographic and Work Characteristics

Medical surgical nurses were described according to age, gender, marital status, highest educational attainment, level of hospital, years of experience in current assignment, and monthly salary.

Knowledge on Nursing Documentation

The knowledge part consisted of 10 statements that can be answered by “Yes” (1 point) or “No” (zero). Highest and lowest scores can be 10 and 0, respectively. Those who scored at least six points (55%-60%) had good knowledge, while a score of 5 points or less suggested poor knowledge of nursing documentation. It has been found to have good internal consistency and reliability based on Cronbach’s alpha score of 0.71 [6].

Attitude on Nursing Documentation

A 5-point Likert scale was used to determine the nurses’ attitude towards documentation. Responses ranged from strongly disagree (1) to strongly agree (5). Higher mean scores suggested a favorable attitude. Using a mean score as reference, those who scored greater than or equal to the mean score of total attitude questions were classified as having a favorable attitude, and those who scored less than the mean score were considered to have an unfavorable attitude towards nursing care documentation [5]. The internal consistency and reliability of attitude towards nursing care documentation is good, based on Cronbach’s alpha score of 0.83 [6].

Organizational Factors at Work on Nursing Documentation

There are 10 questions in this section, which are mostly answered by a “Yes” or “No” response. The statements are organizational in nature as they apply to the documentation of nurses. This section of the questionnaire serves to describe the nurses in terms of how the organizational factors influence their documentation [1,5].

Documentation Practice

The documentation practice is composed of nine statements that can be answered as never (0), sometimes (1), or always (2). This self-reported practice of documentation covers information about a patient’s health status, nursing needs, nursing care, and response to nursing care. With the highest and lowest possible scores of 27 and 9 points, respectively, nurses can be described as having good (equal to or more than the cut-off median score) or poor (less than the cut-off median score) documentation practice [5]. The internal consistency and reliability of this questionnaire are good, with Cronbach’s alpha scores of 0.888 and 0.74 based on studies [2,6].

Specific procedures based on study objectives

After the ethical clearance certificate was provided, the researcher formulated communication letters addressed to the Chief Nursing Officer (CNO) of each hospital and to the management head for research in each locale. The letter discussed the study’s purpose, required participation of the respondents, plans for communication of future findings, and emphasis on data privacy and confidentiality.

The next step involved the preparation of the research instruments. Since the knowledge, attitude, and practices of nursing documentation are in an open-access nature, the researcher informed the author of the instrument for reference and courtesy purposes. Finalization of selected sociodemographic, clinical, and organizational characteristics was carried out after an extensive literature review. The electronic version was created and pilot tested to ensure that all required details in the electronic version were accurate, complete, and retrieved following data entry.

A list of the respondents’ names and email addresses was requested from the management. Unfortunately, due to concerns of privacy, the link to the questionnaire was sent instead to the staff portal for participation. The respondents were given at least one week to complete the online survey. 

Ethical considerations

An ethical clearance certificate was secured from the Angeles University Foundation’s Ethics Review Committee (ERC) before conducting the study. The electronic version of the questionnaire started with providing the necessary information that pertained to the informed consent before the respondents were directed to the specific details required for the study. The informed consent detailed the purpose of the study, respondent’s information, the nature of involvement, possible benefits, hazards and problems, related illness or injury clauses, voluntary consent, secrecy undertaking, and protection of data and contact information of the researcher and AUF-ERC panel. Because the study was conducted as a graduate research requirement at Angeles University Foundation, ethical clearance was obtained through the university’s ERC, data collection proceeded only after written administrative permissions from each participating Texas hospital, and the absence of a U.S.-based IRB review is acknowledged as a study limitation.

Ethical considerations were taken seriously in the conduct of the study, with emphasis on informed consent from all respondents. This was to ensure that the respondents were fully aware of the study’s content and purpose, and that they could voluntarily agree to use their data for research. They also had the right to withdraw from participating in the survey at any time, and they could not be forced to complete the answers. Information provided by respondents was used only for this study and was not disclosed or used for other purposes. 

The risks and inconveniences were considered minimal for this study. The electronic version of the study was sent through a link for the respondent to complete for about 10 to 15 minutes. Should the respondents feel uncomfortable during the period of data collection, the respondents have the right not to complete the questionnaire. As part of their ethical duty to the respondents, they were informed of any potential harm or discomfort that may come to them. As this may require time from them to answer the questionnaire, information about the required length for participation was provided. If some areas of the questionnaire caused them minor emotional risk, such as personal information, the researcher clarified confidentiality policies. 

The study is beneficial to nurses as documentation is an important legal responsibility. Understanding the factors that correlate with good or poor documentation practice can serve as a reference for improvement.

The information gathered in the study was kept confidential. All soft copies of files were password-protected, and the password was only known to the researcher, adviser, and analyst. The data was stored for a period of one year from the time of completion of the research process. Disposal of collected data followed the retention period set for this study.

Statistical analysis of data 

To describe the respondents’ sociodemographic, work, organizational characteristics, knowledge, attitude, and practice of electronic nursing documentation, descriptive statistical tests were used. These came in the form of mean and standard deviation for continuous variables, and frequency and percentage distribution for categorical data.

Correlational tests (e.g., Spearman rho) described the correlation of sociodemographic, work, and organizational characteristics of the respondents towards their documentation practice. Knowledge and attitude towards nursing documentation were also correlated with nurses’ electronic documentation practice. Linear regression, another form of correlational test, was also used to determine how these selected factors predict the likelihood of poor or good documentation practice among nurses.

## Results

Nurses’ socio-demographic and work characteristics

Table 1 shows the 379 nurses’ socio-demographic and work characteristics. They are mostly between 38 and 39 years old (mean = 38.17, SD = ±9.93), female (244, 64.4%), married (186, 49.1%), and bachelor’s degree holders (289, 76.3%). They work mostly in a general hospital (254, 67%) in the medical ward (290, 76.%) for a period between 9 and 10 years (mean 9.77, SD=±8.51) with annual gross income ranging from 80,000 to 90,000 USD (86, 22.7%).

**Table 1:** Nurses' socio-demographic and work characteristics %: percentage; f: frequency; N: 379; SD: standard deviation

Socio-demographic and work characteristics	
Age: mean (SD±)	38.17 (9.93)
Gender	
Male: f (%)	135 (35.6)
Female: f (%)	244 (64.4)
Marital status	
Single	162 (42.7)
Married	186 (49.1)
Widowed	10 (2.6)
Divorced	21 (5.5)
Highest educational attainment	
Associate: f (%)	24 (6.3)
Bachelor’s: f (%)	289 (76.3)
Master’s: f (%)	66 (17.4)
Level of hospital when currently working	
General: f (%)	254 (67)
Specialized: f (%)	125 (33)
Hospital area currently assigned	
Medical: f (%)	290 (76.5)
Surgical: f (%)	89 (23.5)
Hospital where currently working	
A: f (%)	131 (34.6)
B: f (%)	90 (23.7)
C: f (%)	52 (13.7)
D: f (%)	106 (28)
Years of experience in current work: mean (SD±)	9.77 (8.51)
Annual gross income	
Less than 60,000 USD: f (%)	38 (10)
60,001 to 70,000 USD: f (%)	77 (20.3)
70,001 to 80,000 USD: f (%)	60 (15.8)
80,001 to 90,000 USD: f (%)	86 (22.7)
90,001 to 100,000 USD: f (%)	61 (16.1)
100,001 to 110,00 USD: f (%)	40 (10.6)
110,001 to 120,000 USD: f (%)	14 (3.7)
More than 120,000 USD: f (%)	3 (0.8)

Nurses’ knowledge of electronic documentation

The knowledge of nurses on electronic documentation is presented in Table 2. All the nurses demonstrate good knowledge of documentation (mean= 9.88, SD=±.371) that which serves to communicate pertinent information such as assessment and outcome evaluation of patients among health staff, ensures legal protection, and recording to technical tasks like medication administration. While more than 95% of these nurses are aware of electronic documentation importance, some do not consider it a professional responsibility (4, 1.1%), which can lead to severe injury or even death of a client (21, 5.5%). They are not knowledgeable with operational standards involved in documentation (4, 0.8%), including the accurate and timely recording (4, 1.1%) and avoidance of non-standardized abbreviations to prevent error and confusion (4, 1.1%)

**Table 2:** Nurses' knowledge of electronic documentation %: percentage; f: frequency; N: 379; SD: standard deviation

Statements	Yes f (%)	No f (%)
Documentation of patients’ care is part of professional responsibilities.	375 (98.9)	4 (1.1)
Error-free, complete, easily readable, and chronological documentation are some of the principles that need to be followed while documenting nursing care activities.	376 (99.2)	3 (0.8)
Patient care documentation is helpful to improve the quality of care, for better communication with health care staff, for education and research, and for legal protection and health planning.	379 (100)	0 (0)
Assessment data, the progress of patients, transfer and discharge of patients, care provided, and evaluation of outcomes are the main nursing activities a nurse is expected to document.	379 (100)	0 (0)
Inadequate documentation of nursing care can lead to severe injury or the death of a client and poor development of the nursing profession.	358 (94.5)	21 (5.5)
Using non-standard abbreviations when documenting patient care can lead to errors, waste of time, and confusion.	375 (98.9)	4 (1.1)
The nurse should adhere to an operational standard for nursing care documentation when documenting care provided for a patient.	375 (98.9)	4 (1.1)
Components of documenting medication administration include the names of medications, date and time of medications administered, routes and dosage of medications administered, and the nurse's name and signature.	379 (100)	0 (0)
The same nurse who provided the care or a colleague who assisted with the care is responsible for documenting the care provided to a patient.	376 (99.2)	3 (0.8)
Documenting the date and time of care, recording only what the nurse saw or did, recording in chronological order, putting single lines, making corrections clearly, and recording frequently are the main actions that protect a nurse from legal suit.	375 (98.9)	4 (1.1)
Mean (SD±)	9.88 (.371)	
Classification of knowledge on electronic documentation		
Good: f (%)	379 (100)	
Poor: f (%)	0 (0)	

Nurses’ attitudes toward electronic documentation

The attitude of nurses on electronic documentation is shown in Table 3. The majority of the nurses have a favorable attitude toward electronic documentation (369, 97.4%). Among those with reported highest mean scores, nurses agree that electronic documentation relates to providing legal protection (mean=4.66, SD=±.67), contributing to patient safety (mean= 4.53, SD=±.84), reflection of actual care provided (mean= 4.57, SD=±.72), and a need for sufficient knowledge on how to record accurately (mean=4.57, SD=.71) even during challenges at work (mean= 4.50, SD=±.77). Lower mean scores associate some nurses to disagree on areas about timely admission assessment to be completed within the first hour (mean= 4.25, SD=±.88), patients being able to know what nurses record in their chart (mean= 4.22, SD=±.99), and a well-written report to replace oral shift report (mean=4.14, SD=±1.11). Ten nurses (2.6%) were found to have a non-favorable attitude about electronic documentation.

**Table 3:** Nurses' attitude toward electronic documentation %: percentage; f: frequency; N: 379; SD: standard deviation

Statements	Mean (SD±)
Nursing documentation helps to create good nurse-to-patient relationships	4.34 (.94)
Quality documentation of nursing care can add value to my hospital	4.51 (.82)
Proper documentation has a positive impact on patient safety	4.53 (.84)
Although challenges are known to exist, I am expected to do complete and accurate documentation	4.50 (.77)
A well-written report can replace an oral shift report	4.14 (1.11)
Documented care is just as important as the actual care	4.57 (.72)
Nursing notes are meaningful and give me legal protection	4.66 (.67)
Patients should know what we are documenting in their chart	4.22 (.99)
Nurses have sufficient knowledge of the documentation procedure	4.57 (.71)
Nursing admission assessment should be completed within 1 hour	4.25 (.88)
Mean (SD±)	4.43 (.67)
Classification of attitude on electronic documentation	
Favorable: f (%)	369 (97.4)
Non-favorable: f (%)	10 (2.6)

Nurses’ organizational factors at work on electronic documentation

The organizational factors at work are presented in Table 4. Surrounding electronic documentation, most of the nurses receive in-service training (348, 91.8%) and are aware of operational standards for both nursing documentation (356, 93.9%) and nursing care (351, 92.6%), with materials readily available (358, 94.5%). Supervisors also follow up (347, 916%), and there is a monitoring system in place (353, 93.1%) to check on the quality of documentation. Notably, there are some nurses who do not have enough time to document provided care for clients (79, 20.8%), have not received motivation from supervisors (64, 16.9%), and have been exhausted during patient care, which prevents accurate documentation (318, 83.9%).

**Table 4:** Nurses' organizational factors at work on electronic documentation %: percentage; f: frequency; N: 379

Organizational factors	Yes f (%)	No f (%)
Have you received any in-service training about nursing care documentation?	348 (91.8)	31 (8.2)
Do you have enough time to document the nursing care provided for clients?	300 (79.2)	79 (20.8)
Is there an operational standard for nursing documentation in your hospital?	356 (93.9)	23 (6.1)
Are you familiar with the operational standard of nursing care?	351 (92.6)	28 (7.4)
Availability of nursing care materials for documentation	358 (94.5)	21 (5.5)
Have you received any motivation from the supervisor of your hospital?	315 (83.1)	64 (16.9)
Are there any obligation and follow-up to document nursing care activities from your hospital?	347 (91.6)	32 (8.4)
Is there a monitoring and evaluation (M&E) system for nursing documentation in your hospital?	353 (93.1)	26 (6.9)
What is the average number of patients cared for by you per shift?	5 or less pts 183 (48.3)	> 5 pts 196 (51.7)
Do you feel any fatigue or exhaustion during patient care that prevents you from documenting nursing care activities?	318 (83.9)	61 (16.1)

Nurses’ electronic documentation practices

In Table 5, it is shown that all nurses (379, 100%) demonstrate good practice of electronic documentation (mean 25.36, SD=±2.35). At most, they always document assessment findings (346, 91.3%), and interventions (347, 91.6%) such as administered fluid (343, 90.5%), and medication (353, 93.1%) for every patient. While still most of them have constant good practice, there are some nurses who do not constantly (sometimes) document problems (92, 24.3%), response to intervention (74, 19.5%), and education (110, 29%) to every patient. 

**Table 5:** Nurses' electronic documentation practices %: percentage; f: frequency; N: 379; SD: standard deviation

Practices	Always f (%)	Sometimes f (%)	Never f (%)
Do you document the assessments you have done for every patient?	346 (91.3)	33 (8.7)	0 (0)
Do you document problems you find (the nursing diagnosis) for every patient?	287 (75.7)	92 (24.3)	0 (0)
Do you document the interventions you have done for every patient?	347 (91.6)	32 (8.4)	0 (0)
Do you document the response to your intervention for every patient?	305 (80.5)	74 (19.5)	0 (0)
Do you document the fluid you administer to every patient?	343 (90.5)	36 (9.5)	0 (0)
Do you document the medication you administer to every patient?	353 (93.1)	26 (6.9)	0 (0)
Do you document the education or advice you have provided to a patient?	269 (71)	110 (29)	0 (0)
Do you document the fluid balance status of the patient?	294 (77.6)	79 (20.8)	6 (1.6)
Are all your documentations done immediately after care is provided to the patient?	264 (69.7)	106 (28)	9 (2.4)
Mean (SD±)	25.36 (2.35)		
Classification of documentation practices			
Good: f (%)	379 (100)		
Poor: f (%)	0 (0)		

Relationship of nurses' socio-demographic, knowledge, attitude, and organizational factors toward electronic documentation practice

Table 6 reveals that age, gender, level and area of hospital, years of current work experience, annual gross income, and number of assigned patients per shift have no relationship (p> .05, .01) with electronic documentation practices among nurses. 

**Table 6:** Relationship of nurses’ socio-demographic and work characteristics, knowledge, attitude, and organizational factors toward electronic documentation practice H₀: null hypothesis; N: total *Mean comparison using an independent t-test at .05 level of significance **Mean comparison using one-way ANOVA where when the p-value is rejected, multiple comparison is carried out at .05 level of significance

Socio-demographic and work characteristics	p-value	Decision	Remarks
Age	.425	Accept H₀	
Gender*	.234	Accept H₀	
Marital status**	< .001	Reject H₀	Divorced < Single, Married, Widowed
Highest educational attainment**	< .001	Reject H₀	Master’s > Bachelor’s > Associate
Level of hospital when currently working*	.583	Accept H₀	
Hospital area currently assigned**	.913	Accept H₀	
Years of experience in current work	.281	Accept H₀	
Annual gross income**	.159	Accept H₀	
Knowledge	< .001	Reject H₀	Coefficient= .287 (positive low correlation)
Attitude	< .001	Reject H₀	Coefficient= .682 (positive substantial correlation)
Organizational factors*			
… received any in-service training about nursing care documentation?	< .001	Reject H₀	Yes > No
… have enough time to document the nursing care provided for clients?	< .001	Reject H₀	Yes > No
… have operational standard for nursing documentation in your hospital?	< .001	Reject H₀	Yes > No
… familiar with the operational standard of nursing care?	< .001	Reject H₀	Yes > No
… Availability of nursing care materials for documentation	< .001	Reject H₀	Yes > No
… received any motivation from the supervisor of your hospital?	< .001	Reject H₀	Yes > No
… have any obligation and follow-up to document nursing care activities from your hospital?	< .001	Reject H₀	Yes > No
… have a monitoring and evaluation (M&E) system for nursing documentation in your hospital?	< .001	Reject H₀	Yes > No
… average number of patients cared for by you per shift?	.122	Accept H₀	
… feel any fatigue or exhaustion during patient care that prevents you from documenting nursing care activities?	< .001	Reject H₀	No > Yes

Interestingly, marital status (p <.001) has been associated with documentation practice where single, married, and widowed nurses have consistently better practices when compared to divorced nurses. Those with a master’s degree have better documentation practices (p < .001) when compared to bachelor’s and associate degree holders. Similarly, better knowledge and attitude of electronic nursing documentation are associated with better practices (p < .001) with positive low and positive substantial correlation, respectively. 

Consistently, those who receive in-service training, are aware of operational standards, and monitoring systems in place and other available materials, have adequate time for documentation, and are motivated by supervisors with follow-up tend to have better electronic documentation practices (p< .001). Exhausted nurses tend to have poorer documentation practice scores (p< .001).

Meanwhile, Table 7 shows a regression analysis to determine whether knowledge and attitude can be associated with documentation practice, as an outcome. It also explains the amount of association with respect to changes in knowledge and attitude scores toward documentation practice scores.

**Table 7:** Knowledge and attitude in association with electronic documentation practice

Predictor	Value	Decision/remarks
Knowledge		
p-value	< .001	The model examining the association between knowledge and documentation practice is statistically significant.
R	.233	Weak positive association between knowledge score and documentation practice.
R square	.054	Knowledge accounts for 5.4% of the variance in documentation practice scores.
Slope B	1.475	For each 1-point increase in the knowledge score, the documentation score increases by 1.475 points on average.
Attitude		
p-value	< .001	Reject H₀. The regression model significantly predicts the documentation practice score.
R	.527	Moderate positive relationship
R square	.227	About 27.7% of the variance in documentation practice score is explained by the attitude score
Slope B	1.843	For each 1-point increase in the attitude score, the documentation score increases by 1.843 points in average.

The first section of the regression model shows a weak positive correlation between knowledge and documentation practice of nurses (p < .001). Only 5.4% (R square = .054) of the variance in total documentation practice score is explained by the knowledge score of nurses. For each 1-point increase in knowledge scores, 1.475 points are increased toward documentation practice scores.

Additionally, the second section of the regression model is also statistically significant, which states that the attitude of nurses has a moderate positive relationship with documentation practices. About 27.7% (R square = .277) of the variance in documentation practice score is associated with the scores obtained by nurses in attitude. This also means that about 27.7% of the differences in practice scores between nurses can be explained by their attitude scores. Specifically, a one-point increase in the attitude score can increase the documentation practice score by 1.843 points. In a nutshell, nurses with a better attitude tend to practice better in documentation. While attitude explains some of the differences in how much nurses practice, the strength of the association between attitude and documentation practice is statistically stronger when compared to knowledge scores.

## Discussion

Grounded on the results of this study involving 379 nurses, it affirms that nurses are highly knowledgeable and demonstrate a favorable attitude and good practices toward electronic documentation. It also emphasizes organizational support, educational level, and managerial support as capable of promoting quality documentation. Fatigue and lack of time were identified as key barriers to poor documentation practices. Furthermore, age, gender, hospital setting, income, and patient load did not show a significant correlation with documentation practices.

The discussion is divided into socio-demographic and work characteristics, knowledge, attitude, organizational factors at work, and practices in electronic documentation. It also highlights the role of these variables in nurses’ electronic documentation practices.

Nurses’ socio-demographic and work characteristics, knowledge, and attitude toward electronic documentation

Irrespective of the inclusion criteria, nurses working only in the medical and surgical wards were included. Predominantly working in medical wards, these nurses spent around 9 to 10 years in the workplace with annual income closely comparable with other states’ expected income in the country. 

Nursing documentation is a key factor in workflow [16,17], and education about improving the nurses' knowledge of documentation is essential [18]. This study highlights a universally high knowledge level about electronic documentation, specifically in roles related to communication, legal protection, and patient safety. It is rare to see 100% of nurses in a sample classified as having good knowledge, and 97.4% of the nurses to have a favorable attitude toward electronic documentation. Findings from previous studies showed 42% of nurses with documentation practice [5] and 37.74% of the nurses with good knowledge of documentation [19].

While presented in small proportions, it is essential to note that some nurses were not aware of the implications of poor documentation in matters related to patient safety, operational standards, and accurate documentation practices. Nursing standards in electronic documentation contribute to continuity of care and patient safety. Having sufficient knowledge of the importance of electronic documentation results in better practices [20,21]. In a study, graduate nurses demonstrated an adequate knowledge base of documentation, suggesting that training and exposure are provided to them [22]. Since computer efficacy is required for electronic documentation, in-service training is required to meet documentation requirements, especially for those who are new to the workplace.

A favorable attitude toward electronic documentation has been highlighted in this study. Similar to knowledge, legal protection is emphasized by most agree by nurses in this study. Safety has been marketed as a strong advantage for healthcare facilities [23]. Electronic documentation safeguards legal protection by reducing medical errors through the quality and efficiency of information sharing about patients cared for [24,25]. It also uncovers the idea that documented action reflects provided care to enhance patient safety. Another study emphasizes the role of electronic documentation toward patient safety, a cornerstone of professional nursing practice [23]. Through recording, essential information is communicated, which sets a uniform page for the care provided to respective patients. Similarly, documentation is a key source of clinical information in meeting both professional and legal standpoints [5]. 

While still positive, lower rated items encompass the timeliness of admission assessment and whether a written report can become a substitute for oral shift handoff. Timely documentation is also a principle in documentation, as it does not compromise the accuracy of recorded data.

Nurses’ organizational factors at work on electronic documentation

In this study, the organizational factors affecting electronic documentation show strong support of the hospitals through in-service training, available monitoring systems, standards, documentation materials, and supervisory follow-up. A combination of teaching through modeling and understanding charting to standard requirements impacts the development of competency in electronic documentation [22]. Similarly, nurses who are trained and have established computer self-efficacy have noted improved perception and attitude toward documentation [26]. Nurse leaders need to be engaged to safeguard the implementation of relevant nursing standards toward documentation [21]. 

However, the majority of the nurses reported fatigue and exhaustion, and some nurses indicated a lack of time to complete electronic documentation. Additionally, reports indicated that some nurses did not have enough motivation or encouragement from their immediate supervisors. It has been reported that nurses can benefit from electronic documentation when its system does not consume a significant amount of work time, and this can be achieved when redundancy in charting is avoided [23]. Since documentation systems are customized for every organization, receiving in-service training is imperative so the nurses can use the system consistently and effectively [22]. 

Nurses’ electronic documentation practice

This study portrays that all nurses were classified as having good practice in electronic documentation. Practices include recording of administered medications, implemented interventions, and identified assessments. The results of this study are confirmed by other studies [27,28]. Fewer areas of documentation encompass nursing diagnoses, response to interventions, and patient education. It is also noted that only roughly around 7 out of 10 nurses documented immediately after care, which created an impact on documentation accuracy and timeliness.

Relationship of nurses’ socio-demographic and work characteristics, knowledge, and attitude toward electronic documentation practices

This study affirms the role of educational attainment, knowledge, attitude, and organizational factors in the quality of electronic documentation. On the other hand, age, gender, hospital type, ward type, years of experience, income, and number of assigned patients per shift did not show any statistically significant correlation. Interestingly, divorced nurses had significantly lower documentation practice scores when compared to single, married, and widowed counterparts, which may call for further studies to refute or support this claim. This may be a personal or social factor that may be a novel point for further study.

The immense value of knowledge has been supported in this study, which can influence documentation quality. Specifically, those with master’s degrees were found to have better documentation practices than those with bachelor’s or associate degrees, supported by a study that affirms better documentation resulting from better educational attainment [5].

A strong positive correlation emerged between nurses’ knowledge and practices of electronic documentation. Knowledge and attitudes were predictors of better documentation behavior among nurses [16,20]. It is worth noting that a strong correlation of attitude with documentation practice (even stronger than knowledge) exists, which suggests that interventions to promote attitude, such as motivation from supervisors, follow-up with positive reinforcement, and communication of documentation expectations, may generate more practical improvement in documentation practices, in general. 

Strong institutional supports in many respects are mirrored in this study to affect documentation practices. Organizational factors, such as in-service training, operational standards, documentation material availability, and supervisor follow-up up positively affect documentation practice scores [5]. This is also because these factors enhance knowledge and attitude, which were also found to be positively correlated with documentation practices. The availability of in-service training and communication of standards and required expectations are strongly linked with better practice of documentation [16]. It is also worth noting that adequate time and fatigue can influence documentation practices in positive and negative ways, respectively. Time constraint was identified as the key barrier to meeting documentation expectations [5]. Considering that nurses spend 25% of their time on documentation, providing adequate time, training, and follow-up can immensely contribute to better practices [22]. These institutional supports underscore the relevant impact of system-level programs for nurses over just individual interventions. 

While age, years of experience, and type of hospital did not show a significant association with documentation practice in the current study, nurses above 40 years were 2.59 times more likely to practice documentation than those who were between 20 and 29 years old from another study [5]. As they become older, they may recognize documentation benefits which improve their attitude and, in turn, their practices. More years of experience also mean longer years of exposure to documentation practice, which may improve over time. Additionally, those who work in specialized hospitals tend to have 4.2 times better documentation practices than primary hospitals due to more available documentation resources and a requirement for more specialized documentation. 

The study is limited by its self-reported, cross-sectional design, which introduces potential response and social-desirability bias and restricts interpretation to the period of data collection. The sample size was based on estimated rather than exact nurse populations, and the use of proportionate non-probability sampling with unknown response rates constrains representativeness and generalizability; in addition, ceiling effects in several scales further limit the ability to discriminate between respondents. The instruments were adopted from previous studies without formal cultural adaptation, with only pilot usability testing conducted, and differences in hospital software interfaces and unmeasured IT-related factors may also affect documentation practices and the relationships observed.

## Conclusions

The study highlights remarkably high levels of knowledge, a favorable attitude, and strong documentation practices among medical and surgical ward nurses, particularly in communication, legal protection, and patient safety. The critical role of education, organizational support, and operational standards was emphasized to contribute to documentation quality and compliance. However, despite these overall positive results, important gaps were observed, including inconsistent documentation of problems, responses to interventions, patient education, and delays in documenting immediately after care. While nurses’ documentation practices were commendable, barriers such as fatigue, time constraints, and limited supervisor motivation needed to be addressed. Institutional support through in-service training, clear operational standards, and available resources positively and substantially influence documentation behaviors, yet this support must also target the areas where documentation remains inconsistent. As electronic documentation continues to influence modern healthcare, sustaining organizational structures, systems, and processes among nurses is essential to ascertain accurate, timely, and comprehensive patient health records.

## Appendices

Research questionnaire

Section I: Sociodemographic, Clinical, and Organizational Characteristics

Age in years: ____ Gender: ( ) Male, ( ) Female Marital status: ( ) Single, ( ) Married, ( ) Widowed, ( ) Divorced Highest educational attainment: ( ) Associate, ( ) Bachelor, ( ) Master, ( ) Doctorate Level of hospital where you currently work: ( ) Specialized hospital, ( ) Primary hospital Years of experience in current assignment: ___ Your monthly salary: ___

Section II- Documentation Practice

Instruction. Please read the following questions carefully and put (√) on the correct answer option.

**Table 8:** Documentation practice

SN	Items	Never	Sometimes	Always
1	Do you document the assessments you have done for every patient?			
2	Do you document problems you find (the nursing diagnosis) for every patient?			
3	Do you document the intervention you have done for every patient?			
4	Do you document the response to your intervention for every patient?			
5	Do you document the fluid you administered to every patient?			
6	Do you document the medication you administered to every patent?			
7	Do you document the education or advice you have provided to a patient?			
8	Do you document the fluid balance status of the patient?			
9	Is all your documentation done immediately after care provided to the patient?			

Section III: Knowledge of Nursing Documentation

**Table 9:** Knowledge of nursing documentation

SN	Items	Yes	No
1	Documentation of patients’ care is part of professional responsibilities.		
2	Error-free, complete, easily readable, and chronological documentation are some of the principles needed to be followed while documenting nursing care activities.		
3	Patient care documentation is helpful to improve the quality of care, for better communication with health care staff, for education and research, and for legal protection and health planning.		
4	Assessment data, the progress of patients, transfer and discharge of patients, care provided, and evaluation of outcomes are the main nursing activities a nurse is expected to document.		
5	Inadequate documentation of nursing care can lead to severe injury or the death of a client and poor development of the nursing profession.		
6	Using non-standard abbreviations when documenting patient care can lead to errors, waste of time, and confusion.		
7	The nurse should adhere to an operational standard for nursing care documentation when documenting care provided for a patient.		
8	Components of documenting medication administration include the names of medications, date and time of medications administered, routes and dosage of medications administered, and the nurse's name and signature.		
9	The same nurse who provided the care or a colleague who assisted with the care is responsible for documenting the care provided to a patient.		
10	Documenting the date and time of care, recording only what the nurse saw or did, recording in chronological order, putting single lines, making corrections clearly, and recording frequently are the main actions that protect a nurse from legal suit.		

Instruction: Please read the following questions carefully and put (√) on the correct answer option.

Section IV: Attitude of Nursing Documentation

Instruction: There are statements about the attitude of nursing documentation among nurses and each statement has five alternatives with a five-point scale. 1= strongly disagree, 2= Disagree, 3= neither agree nor disagree (neutral), 4= Agree, 5= Strongly Agree

**Table 10:** Attitude of nursing documentation

SN	Statement	Strongly disagree	Disagree	Neutral	Agree	Strongly agree
1	Nursing documentation helps to create good nurse-to-patient relationships	1	2	3	4	5
2	Quality documentation of nursing care can add value to my hospital	1	2	3	4	5
3	Proper documentation has a positive impact on patient safety	1	2	3	4	5
4	Although challenges are known to exist, I am expected to do complete and accurate documentation	1	2	3	4	5
5	A well-written report can replace an oral shift report	1	2	3	4	5
6	Documented care is just as important as the actual care	1	2	3	4	5
7	Nursing notes are meaningful and give me legal protection	1	2	3	4	5
8	Patients should know what we are documenting in their chart	1	2	3	4	5
9	Nurses have sufficient knowledge of the documentation procedure	1	2	3	4	5
10	Nursing admission assessment should be completed within 1 hour	1	2	3	4	5

Section V: Organizational Factors Affecting the Practice of Documentation among Nurses

**Table 11:** Organizational factors affecting the practice of documentation among nurses

SN	Items	Response
1	Have you received any in-service training about nursing care documentation?	Yes No
2	Do you have enough time to document the nursing care provided for clients?	Yes No
3	Is there an operational standard for nursing documentation in your hospital?	Yes No
4	Are you familiar with the operational standard of nursing care?	Yes No
5	Availability of nursing care materials for documentation	Yes No
6	Have you received any motivation from the supervisor of your hospital?	Yes No
7	Is there any obligation and follow-up to document nursing care activities from your hospital?	Yes No
8	Is there a monitoring and evaluation (M&E) system for nursing documentation in your hospital?	Yes No
9	What is the average number of patients cared for by you per shift?	≤ 2 in ICU or ≤ 6 in other than ICU >2 in ICU or >6 in other than ICU
10	Do you feel any fatigue or exhaustion during patient care that prevents you from documenting nursing care activities?	Yes No
